# Substrate topography-induced osteogenesis of bone marrow stem cells by reducing the chromatin accessibility of YBX1: Substrate topography-induced osteogenesis of BMSCs

**DOI:** 10.3724/abbs.2025065

**Published:** 2025-07-09

**Authors:** Yan Lv, Weishu Dai, Huijing Zhang, Sirui Liu, Mengdie Liu, Xueyan Zhang, Luling Li, Ying Hu, Yi Liu, Lin Song

**Affiliations:** 1 Beijing Institute of Dental Research Beijing Stomatological Hospital Capital Medical University Beijing 100070 China; 2 Department of Periodontology Beijing Stomatological Hospital Capital Medical University Beijing 100070 China; 3 Department of Stomatology Aerospace Central Hospital Beijing 100049 China; 4 The Third Medical Center of PLA General Hospital Beijing 100039 China

**Keywords:** substrate topography, bone marrow mesenchymal stem cell, osteogenesis, chromatin accessibility, YBX1

## Abstract

Stem cell fate is profoundly influenced by a complex interplay of biochemical and biophysical cues, with the latter increasingly recognized for its roles in cellular processes, yet the mechanisms are unclear. Since chromatin accessibility is a critical determinant in the processes of osteogenesis and bone repair, investigating the contributions of open chromatin regions (OCRs) to the intracellular signaling pathways triggered by topographical cues, which lead to osteogenic differentiation is highly valuable. This study explores the impact of the nanotopography of biomaterials on the osteogenic differentiation of human bone marrow stem cells (hBMSCs). By utilizing electrospun poly-L-lactide (PLLA) membranes with random fiber arrangements, we mimic the natural extracellular matrix (ECM) topography to study its effects on hBMSCs, contrasting them with flat PLLA controls. Through high-throughput Assay for Transposase-Accessible Chromatin with sequencing (ATAC-seq) and RNA sequencing (RNA-seq), we reveal that the nanotopography of electrospun surfaces promotes osteogenic differentiation by modulating the chromatin accessibility of the *YBX1* gene promoter, leading to its upregulation. Lentiviral knockdown experiments further confirm the crucial role of YBX1, revealing a reversal of the osteogenic effects induced by nanotopography. This study emphasizes the importance of YBX1 in the osteogenic response to the surface topography of biomaterials and suggests that nanotopographical cues could be harnessed to direct stem cell fate. These findings are important for developing biomaterials that promote specific stem cell outcomes in regenerative medicine. Our results further contribute to a deeper understanding of the mechanisms underlying stem cell differentiation in response to environmental cues and pave the way for the rational design of biomaterials with enhanced osteogenic potential. By elucidating the role of chromatin accessibility and specific transcription factors such as YBX1, this study highlights the intricate interplay between cell-material interactions and the intracellular signaling pathways that govern stem cell fate.

## Introduction

The orchestration of stem cell fate is a multifaceted and intricate process that is significantly influenced by a combination of biochemical and biophysical elements. In recent years, interest among researchers in the realm of biophysical factors, which are omnipresent in the spectrum of biological phenomena, including development, growth, differentiation, immune responses, wound healing, and pathogenesis, has increased. In contrast to their biochemical counterparts, biophysical factors are governed in a manner that closely mirrors the physiological microenvironment, embodying characteristics of sustainability and a more gradual impact. This biomimetic regulation aligns more closely with the physiological microenvironment, thus potentially offering a more nuanced approach to understanding and manipulating stem cell behavior [ 1– 3].

Among many physical properties, the topological cues of biomaterial surfaces, which can regulate stem cell fate through cell-substrate interactions, have gained attention in recent years [ 4– 6]. However, although its effect on stem cell differentiation has been demonstrated, the underlying mechanisms of how topological cues regulate stem cell fate and how stem cells convert perceived topological signals into biochemical signals to regulate their own behaviors are not yet fully understood.

Current studies have focused on the cell membrane and cytoplasm, and it is generally accepted that cellular mechanotransduction processes are transmitted from the outside to the cytoplasm through integrins, focal adhesion, and mechanically gated ion channels [7], *e*. *g*., PIEZO [ 8, 9], which affect the inwards flow of ions, cytoskeletal microfilaments, and microtubule assembly [10]. Despite these advancements, the precise pathways through which signals traverse from the cytoplasm to the nucleus, thereby modulating gene expression and steering cell fate, remain enigmatic.

The cytoskeleton forms an intricate network that connects the cell membrane to the nucleus via the linker of the nucleoskeleton and cytoskeleton complex. This connection facilitates the direct transmission of mechanical signals from the cell’s exterior to its interior. These signals can induce alterations in the 3D structure and accessibility of chromatin, leading to changes in the state of euchromatin and heterochromatin, which in turn affects the expressions of genes [11].

In the present study, we used electrospun PLLA membranes with a random fiber arrangement (Random) as an artificial topographical substrate model. Cast flat PLLA membranes (Flat) were used as the negative control. Our group reported that the substrate topography could induce the osteogenesis of hBMSCs [ 6, 12]. We employed RNA sequencing to explore gene expression, while an assay for transposase-accessible chromatin with high-throughput sequencing (ATAC-seq) was used to identify OCRs, including promoters, enhancers, and transcription factor (TF)-binding sites. By leveraging these findings, we investigated the effects of substrate topography on chromatin accessibility and the subsequent modulation of target gene expression in hBMSCs. We found that the topography of the electrostatic spinning surface promoted the osteogenic differentiation of hBMSCs by altering the chromatin accessibility of the promoter region of Y-box binding protein 1 (YBX1) and increasing YBX1 expression. This study provides a deeper understanding of how physical cues from the microenvironment can direct stem cell fate and has implications for tissue engineering and regenerative medicine.

## Materials and Methods

### Preparation of electrospun PLLA nanofibers

First, 0.7 g of PLLA powder (Shandong Academy of Pharmaceutical Sciences, Jinan, China) was introduced into a solution of 10 mL trifluoroethanol and agitated continuously to ensure complete dissolution. The solution was then extruded from a syringe in a standard electrospinning device (Elite; Ucalery, Beijing, China), which was facilitated by a programmable pump. Using a 23-gauge metal injection needle, the injection pump controls the flow rate at an injection speed of 0.7 mL/h, controlling the diameter of the spinning (200–250 nm). In addition, a constant high-voltage supply of 16 kilovolts was applied to the needle tip to eject the fluid, whereas a metal plate positioned 15 cm from the needle served as the collection surface. The electrospinning process was allowed to continue for 7 h to yield a uniform layer of randomly oriented PLLA nanofibers (Random). For the creation of flat PLLA films (Flat), the polymer solution was spread uniformly onto a flat glass substrate to a specified thickness of 75 μm and air-dried at a temperature of 40°C for a period of 6 h. All these samples (PLLA nanofibers and flat films) were kept in a vacuum oven (DZF-6210; Bluepard, Changzhou, China) at room temperature for 2 weeks to remove residual solvent. The nanostructure of the films was subsequently visualized with a scanning electron microscope (SEM) (SU-8010; Hitachi, Tokyo, Japan) at a voltage of 15 kV [13].

### Cell culture and seeding on PLLA nanofilms

hBMSCs and the culture medium utilized in this study were provided by Cyagen Biosciences, Inc. (Suzhou, China). The medium was changed every 2 to 3 days to maintain optimal cell growth conditions. Once the cells reached 80%–90% confluence, they were harvested with 0.25% trypsin/EDTA solution (Gibico, Carlsbad, USA) and sub-cultured at a density of 6 × 10 ^5^ cells per T75 flask (Nest, Wuxi, China) or 10 ^5^ cells per well in six-well plates (Nest). Cells at the fourth passage were used in this study. The PLLA nanofiber scaffolds and flat films were cut into 4 cm × 4 cm sections and affixed to the caps of centrifuge tubes. The materials were sterilized via radiation for 12 h, followed by thorough washing with phosphate-buffered saline (PBS; Nest).

### Cell morphology and cytoskeletal remodeling

The morphological alterations and cytoskeletal restructuring of hBMSCs were examined using a confocal microscope (TCS SP8; Leica, Wetzlar, Germany). The cells cultured on the materials for intervals of 6 h, 12 h, and 48 h were initially washed with preheated PBS at 37°C and fixed in 4% paraformaldehyde solution (Solarbio, Beijing, China). After being washed twice with PBS, the samples were permeated with 0.1% Triton X-100 (Solarbio) for 10 min. Following a blocking step with 5% BSA (Solarbio) for 60 min, both flat and random PLLA membranes were stained with 100 nM TRITC phalloidin (Solarbio) and counterstained with 10 μg/mL DAPI (Solarbio). Finally, the samples were examined with a confocal laser scanning microscope (Leica).

### RT-qPCR

Total RNA was extracted from cells cultured on PLLA nanofilms with TRIzol reagent (Invitrogen, Carlsbad, USA). Complementary DNA (cDNA) was synthesized from mRNA using the PrimeScript® RT Reagent kit (TaKaRa, Dalian, China) following the manufacturer’s instructions. The cDNA samples were analyzed by RT-qPCR to measure the transcriptional levels of the *RUNX2*, *OCN* and *YBX1* genes. SYBR Green master mix (Roche, Basel, Switzerland) and a 7500 ABI PCR System (Applied Biosystems, Foster City, USA) were used for this purpose. The relative gene expression levels were calculated via the 2 ^–ΔΔCT^ method, and the mRNA data were normalized to the *GAPDH* expression levels for each sample.

The following primer sequences were utilized for real-time RT-PCR: *GAPDH*: 5′-CCTCTGACTTCAACAGCGAC-3′(F)/5′-TCCTCTTGTGCTCTTGCTGG-3′ (R); *RUNX2*: 5′-TCACCTCAGGCATGTCCCTCGGTAT-3′ (F)/5′-TGGCTTCCATCAGCGTCAACACC-3′ (R); *OCN:*5′-CACTCCTCGCCCTATTGGC-3′(F)/5′-CCCTCCTGCTTGGACACAAAG-3′ (R); and *YBX1*: 5′-TGAAGGAGAAAAGGGTGCGG-3′ (F)/5′-TGGTAATTGCGTGGAGGACC-3′ (R).

### RNA sequencing

For RNA sequencing, hBMSCs cultured on the random membrane surface for 2 weeks were designated as the experimental group, while those cultured on the smooth membrane served as the control group. Three wells were set for each group. Sequencing was performed by Frasergen (Wuhan, China). Data analyses included the generation of heatmaps, PCA, and MA plots on the basis of stringent filtering criteria. GO and KEGG enrichment analyses were conducted via hypergeometric distribution methods, with the top 20 significantly enriched terms selected on the basis of Q value thresholds.

### ATAC sequencing

Flat and random groups of hBMSCs were lysed with precooled lysis buffer (Vazyme, Nanjing, China) for 10 min on ice after being rinsed three times in PBS. The cell suspension was centrifuged at 4°C and 500 *g* for 5 min, and the nuclei were collected. The TD501 kit (Vazyme) and the nuclear deposits were equally mixed and placed in a PCR instrument (Vazyme) to perform the transposase interrupt reaction according to a specific procedure. VAHTS DNA Clean Beads (Vazyme) were incubated at room temperature for 30 min and added to the reaction mixture as described above. After being placed on the magnetic frame for 5 min, the magnetic beads (Vazyme) were separated from the liquid. The supernatant was discarded, and 80% ethanol was added for rinsing. The alcohol was discarded after 30 s of incubation. The rinsing was repeated twice, and the mixture was dried with an open lid for 3–5 min. Then, double distilled water was added to cover the magnetic beads. After standing at room temperature for 2 min, the supernatant was carefully collected and transferred to a new EP tube (Nest) to complete the purification of the fragmentation product. The products were amplified by PCR, and the amplified products were length-sorted using VAHTSTM DNA Clean Beads.

ATAC-seq was performed by Frasergen. Bowtie2 software was used to compare the clean data with the reference genome sequence, and the reads included in the comparison were further filtered to remove low-quality comparisons, PCR redundancy, and organelles. Each group sample had two replicates. We checked the insert size distribution of the sequenced fragments to evaluate the ATAC-seq data. Both the size distribution of the inserted fragments and the data reproducibility within the ATAC-seq data were assessed. The TSS enrichment score was estimated to evaluate ATAC-seq data, and the TSS enrichment calculation is a signal-to-noise calculation. If there is a high read signal at TSSs, there should be an increase in the signal up to a peak in the middle. Heatmaps and volcano plots were then produced via log2(fold change) ≥ 0.58 and false discovery rate (FDR) ≤ 0.05 screening.

### Western blot analysis

The cells were lysed via RIPA buffer (P0013B; Beyotime, Shanghai, China) supplemented with protease and phosphatase inhibitors (Halt; Thermo Fisher Scientific, Waltham, USA) to prevent unwanted enzymatic activity. The protein lysates were then collected. Protein concentrations were quantified with a BCA Protein Assay kit (P0012; Beyotime). The lysates were prepared for electrophoresis by mixing with 6× SDS loading buffer (P0015F; Beyotime) at a ratio of 1:5. The samples were denatured at 100°C for 5 min and then loaded onto SDS-polyacrylamide gels for electrophoresis. The resolved proteins were transferred onto PVDF membranes (Millipore, Billerica, USA). The membranes were blocked with 5% non-fat milk (Solarbio) to reduce nonspecific binding and then incubated with primary antibodies specific for β-ACTIN (ab8227; Abcam, Cambridge, UK), OCN (DF12303; Affinity, Cincinnati, USA), and YBX1 (ab76149; Abcam) overnight. The next day, the membranes were incubated with secondary antibodies, either goat anti-rabbit IgG-HRP (ab205718; Abcam) or goat anti-mouse IgG-HRP (SE131; Solarbio), for 2 h. The immunoreactive bands were visualized using an Extremely Sensitive ECL Chemiluminescence kit (P10060; Ncm Biotechnology, Suzhou, China).

### Lentivirus infection

Lentiviral particles, specifically GV493 carrying GFP and shRNA targeting the human *YBX1* gene, were obtained from GeneChem (Shanghai, China). These viruses were used to infect hBMSCs in the presence of polybrene to increase infection efficiency. After a 48-h incubation period, infected hBMSCs were selected for stable integration of the viral genome by culture in medium supplemented with puromycin. The successful knockdown of the target genes was confirmed by qPCR and western blot analysis, which identified and validated the stable clones. The following sequences were utilized for lentivirus infection: *YBX1*-1: 5′-GACGGCAATGAAGAAGATAAA-3′ and *YBX1*-2: 5′-CGTAACCATTATAGACGCTAT-3′. The non-targeting control insert sequence was 5′-TTCTCCGAACGTGTCACGT-3′.

### Statistical analysis

One-way ANOVA and unpaired *t*-tests were used to assess differences among sample groups. Each sample was set up with three biological replicate wells to ensure data reliability and reproducibility. Data analysis was performed using GraphPad Prism 9.0 software, with significance levels of *P*  < 0.05. The results were expressed as the mean ± standard deviation (SD).

## Results

### Topology model to induce osteogenesis of hBMSCs independently

**Figure 1:**
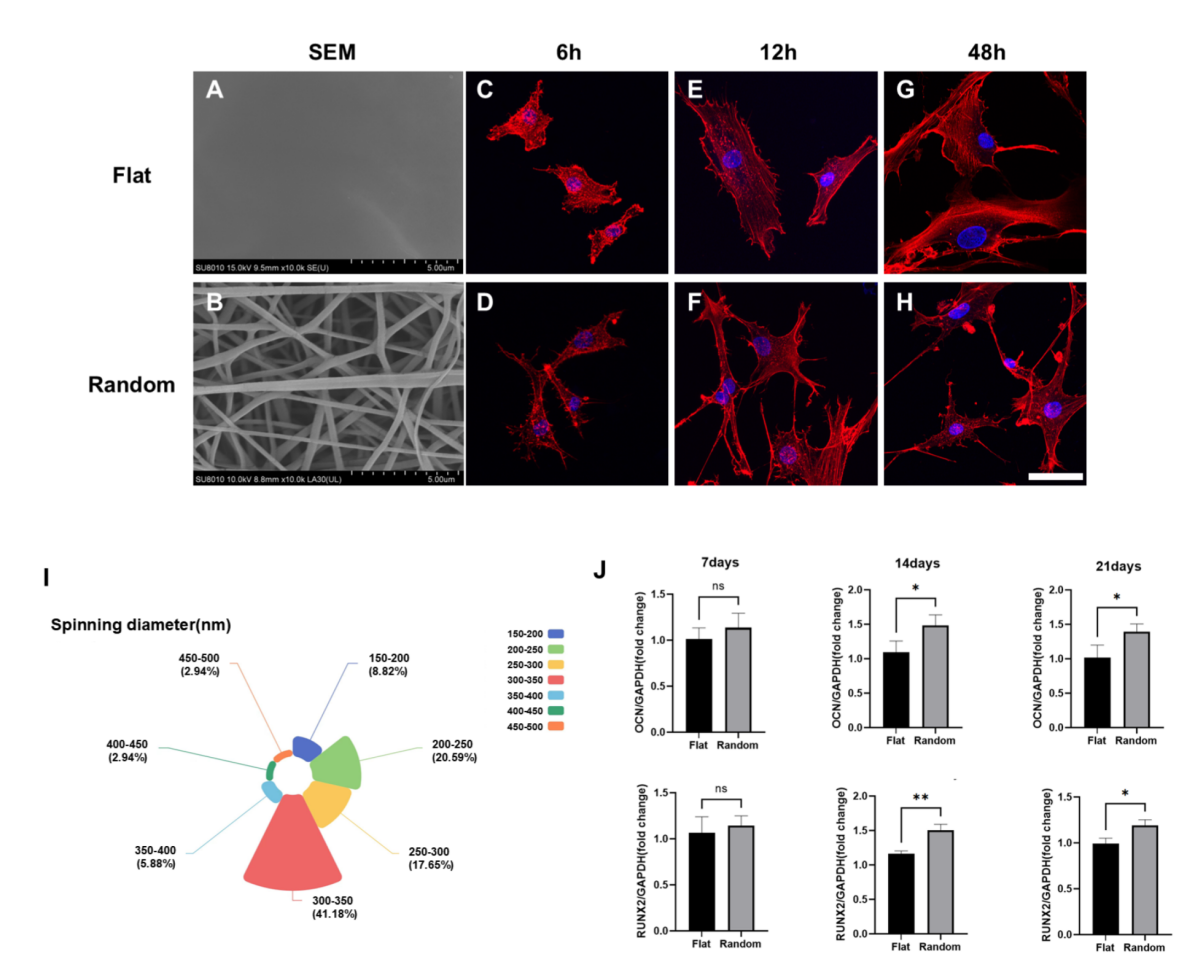
Figure 1 Topology model induces osteogenesis of hBMSCs independently (A,B) SEM images depicting the featureless surface characteristics of the flat PLLA films (A) and randomly arranged nanofibers (B) (scale bar, 5 μm). (C–H) Confocal immunofluorescence staining of nuclei with DAPI (blue) and staining of F-actin by rhodamine-conjugated phalloidin in hBMSCs in the flat group (C,E,G) and the random fiber group (D,F,H) after 6 h (C.D), 12 h (E,F), and 48 h (G,H) of culture. Scale bar, 50 μm. (I) Diameters of nanofibers on the surface of random groups of electrospun membranes. (J) The expressions of OCN and RUNX2 in hBMSCs cultured on both flat and random fibers at 7, 14, and 21 days. Data are presented as the mean ± SD (n = 3). *P < 0.05.

We utilized electrospun PLLA membranes characterized by a random fiber arrangement ( Figure 1B) to emulate the structural characteristics of collagen within the ECM. These membranes were used as artificial substrates. We aimed to investigate how topographical cues influence the regulation of stem cell behavior and the determination of cell lineage fate. For comparative purposes, flat PLLA membranes ( Figure 1A) were employed as a negative control. The diameter of the nanofibers was 292.51 ± 68.50 nm ( Figure 1I). The hBMSCs in the random group presented more, longer, and more robust filopodia, leading to a more elongated and arborized cellular morphology ( Figure 1C–H) and enhanced osteogenic differentiation. The expressions of osteogenic marker genes, such as *RUNX2* and *OCN*, were significantly elevated in the random group, particularly at 14 days and 21 days ( Figure 1J). Our findings suggest that the topological configuration, independent of other factors, is capable of inducing osteogenic morphological changes in hBMSCs and promoting their differentiation along the osteogenic lineage.

### ECM-related processes might be activated mainly in hBMSCs by topographical cues

**Figure 2:**
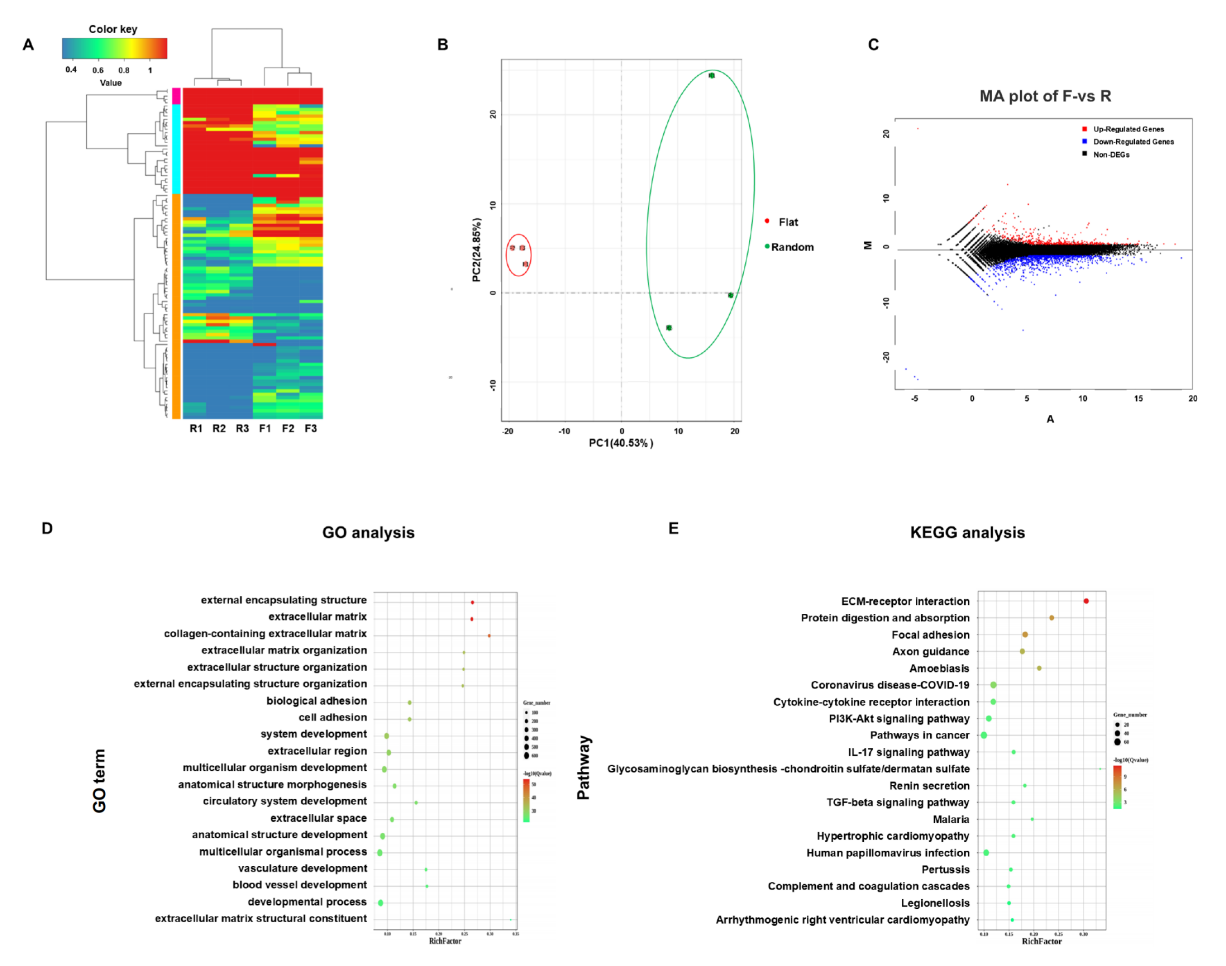
Figure 2 PLLA nanofibrous substrates activate the ECM-receptor interaction signaling pathway via topographical cues (A) Heatmap of hierarchical clustering. (B) Principal component analysis between samples from the flat and random groups. (C) MA map showing the upregulated, downregulated, and unchanged genes in hBMSCs cultured on flat and random substrates for 14 days. (D) GO analysis of significantly enriched pathways related to the ECM and cell adhesion. (E) KEGG analysis of significantly enriched pathways related to ECM-receptor interactions and focal adhesion.

Hierarchical clustering and PCA demonstrated that the topography-induced group was significantly different from the flat group ( Figure 2A,B). Using DESeq2 expression difference analysis, a total of 1510 differential genes (DEGs) were identified, with 451 genes upregulated and 1059 genes downregulated ( Figure 2C). GO analysis revealed that the primary differences between the flat and random groups are in functional categories related to the extracellular matrix and cell adhesion. The top ten terms are external encapsulating structure, extracellular matrix, collagen-containing extracellular matrix, extracellular matrix organization, extracellular structure organization, external encapsulating structure organization, biological adhesion, cell adhesion, system development, and the extracellular region ( Figure 2D). KEGG analysis confirmed that the top pathways are ECM-receptor interaction, protein digestion and absorption, and focal adhesion ( Figure 2E).

### Changes in the chromatin accessibility of the *YBX1* promoter region mediate topographical cues that promote the osteogenic differentiation of hBMSCs

**Figure 3:**
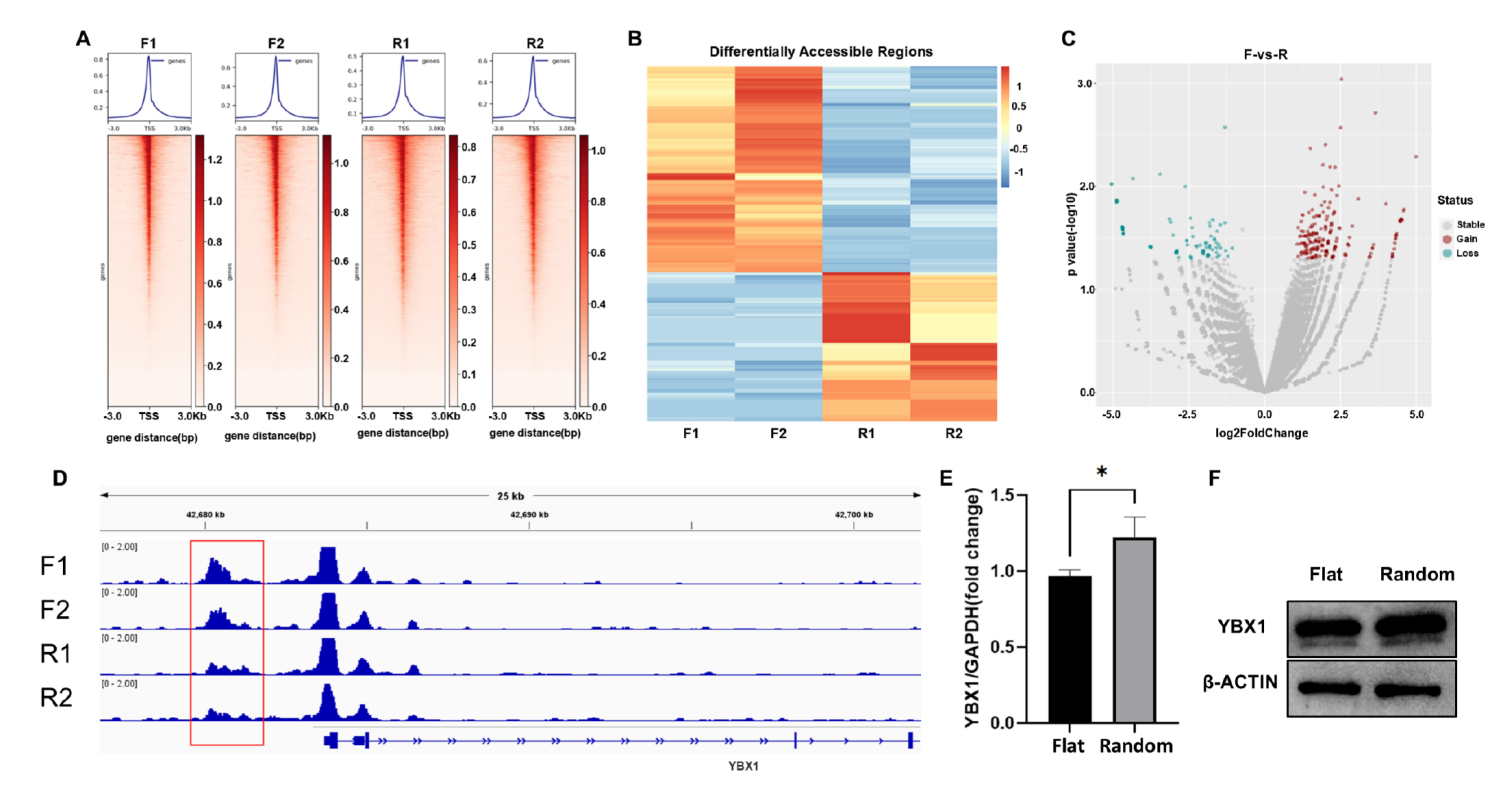
Figure 3 Changes in the chromatin accessibility of the *YBX1* promoter region mediate topographical cues that promote the osteogenic differentiation of hBMSCs (A) Signal enrichment plot of ATAC-seq data in the vicinity of the TSS. (B) Heatmaps clustered the signal values of individual sample DARs between groups. (C) Volcano plot showing the upregulated, downregulated, and unchanged DARs. (D) Chromatin accessibility at the gene loci of the transcription factor YBX1. (E,F) YBX1 mRNA and protein expression levels in hBMSCs in the flat and random groups. Data are presented as the mean ± SD (n = 3). *P < 0.05.

High-throughput sequencing analyses revealed that the surface topography of the materials transmit external physical signals into the cells through the ECM and cell adhesion-related signaling pathways ( Figure 2D,E) and might further transmit signals to the nuclear lamina by the changes in cytoskeleton assembly and arrangement by topographical cues ( Figure 1D,F,H). Nuclear lamina and chromatin interactions at the nuclear periphery promote lamina-associated domain (LAD) maintenance and chromatin compaction, which can affect cell fate [14]. Therefore, we further used ATAC sequencing to detect the chromatin accessibility of hBMSCs on the topography model. We are particularly interested in the binding of transcription factors to the promoter regions of genes, which regulate gene expression, and the specific binding sites need to be investigated further. The results revealed that the reads obtained from ATAC-seq were enriched at the TSS in all the samples ( Figure 3A), which indicated that ATAC-seq library construction was successful and that the sequencing results were reliable. Regions with significant differences in differentially accessible regions (DARs) were detected, and heatmap analysis revealed significantly differentially expressed DARs in the random group and flat group and good clustering within each group of samples ( Figure 3B). The volcano map results revealed 205 regions that were openly upregulated (red) and 148 regions that were openly downregulated (blue) ( Figure 3C). These differences may affect the downstream gene expression regulatory network, thereby influencing stem cell fate, among other factors.

An RNA-seq-ATAC-seq integrated assay was used to analyze potential target genes regulated by chromatin accessibility during the process of hBMSC osteogenic differentiation activated by topological cues. The results revealed that the p34.2 region of chromosome 1 (42,680 kb) was downregulated in terms of chromatin accessibility in the disordered nanofiber topology group, which is located 2 kb upstream of the promoter region of *YBX1* ( Figure 3D), and that the expression of YBX1 was upregulated according to the RNA-seq results. YBX1 expression decreases in senescent hBMSCs and inhibits osteogenic differentiation [15]. qPCR and western blot analysis results revealed that YBX1 expression was upregulated in the random group ( Figure 3E,F). These results indicated that *YBX1* was upregulated by topological activation and might also be a key gene involved in the sensing of changes in surface topography via chromatin accessibility.

### Knockdown of *YBX1* inhibits topography-mediated osteogenic differentiation of hBMSCs

**Figure 4:**
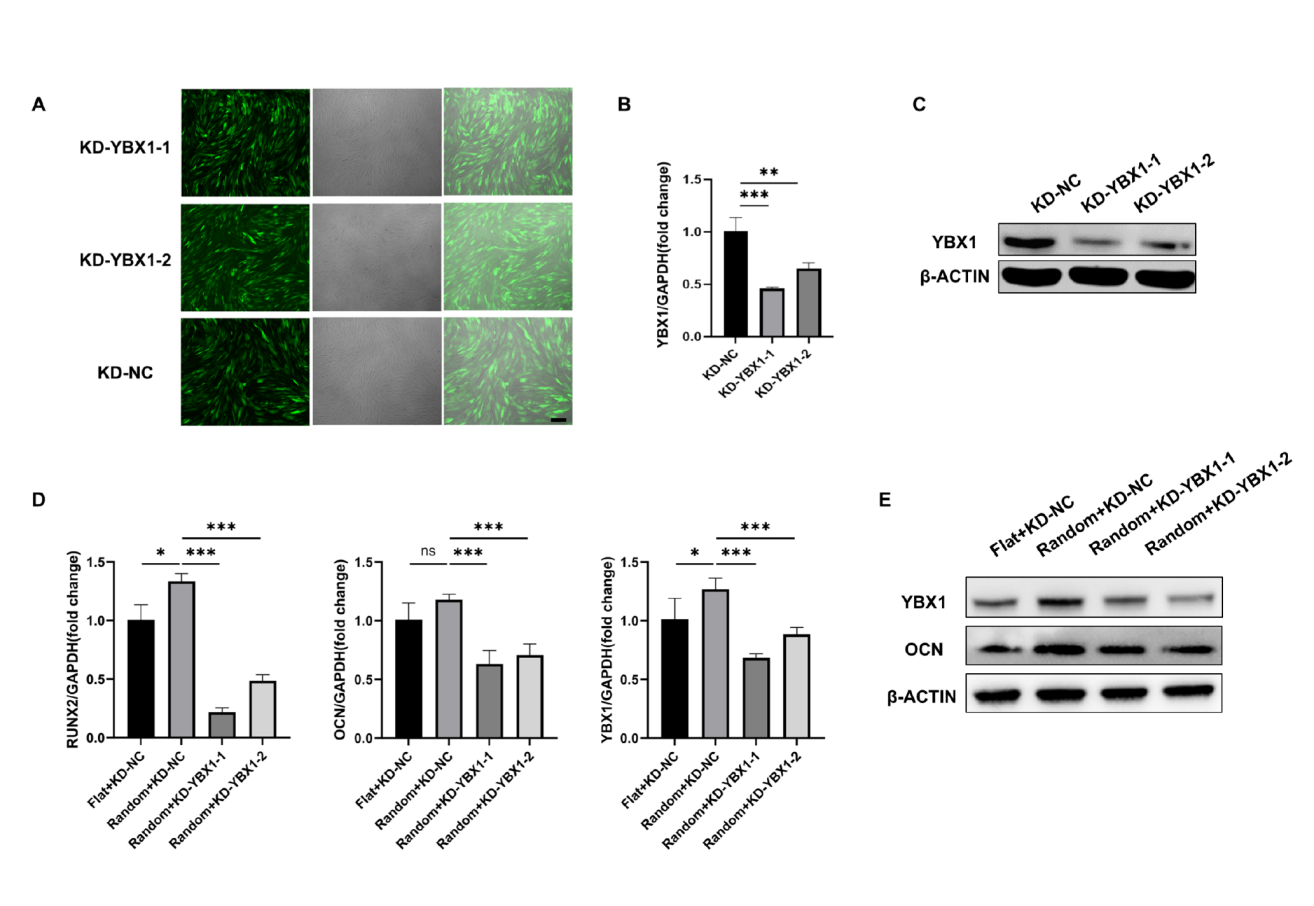
Figure 4 *YBX1* knockdown inhibits topography-mediated osteogenic differentiation of hBMSCs (A) Transfection efficiency of YBX1-specific small hairpin RNA (shRNA) as determined via microscopic observation. Scale bar: 200 μm. (B,C) Transfection efficiency of shRNA determined via qPCR and western blot analysis. (D,E) mRNA and protein levels of YBX1 and osteogenic genes in the flat and random groups of hBMSCs treated with YBX1-shRNA were determined by qPCR and western blot analysis respectively. Data are presented as the mean ± SD (n = 3). *P < 0.05, **P <0.01, ***P < 0.001.

To further define the role of *YBX1*-mediated topographical features in the induction of osteogenic differentiation, a lentivirus was used to downregulate *YBX1* gene expression. Three days after lentiviral transfection, more than 90% of the cells carried green fluorescent protein, which confirmed that lentiviral transduction was successful ( Figure 4A). Furthermore, we performed RT-qPCR and western blot analysis for further validation ( Figure 4B,C). After the hBMSCs were lentivirally stably transfected to knockdown *YBX1*, they were seeded onto two different material surfaces for culture for 14 days. We examined osteoblast-related indicators by RT-qPCR and western blot analysis. The results revealed that the expressions of both OCN and RUNX2 were significantly decreased, indicating that *YBX1* knockdown reversed the ability of the PLLA random nanofibers to induce osteogenic differentiation ( Figure 4D,E).

## Discussion

A good biomimetic structure is the key to ensuring the success of implantation. By studying the mimicry of the ECM physicochemical structure, the biocompatibility of implant materials can be improved, the performance of implant materials can be substantially enhanced, and the healing time can be shortened. The nanotopological cues on the material surface serve as important physical signals that influence the adsorption of proteins and regulate the biological behavior of cells [16]. To clarify the impact of both physical and biochemical signals on cellular regulation, researchers created substrates that were either identical in their physical nanotopography but varied in their biochemical fibronectin (FN) nanopatterns or featured the same FN nanopatterns but exhibited different physical nanotopographies. Their research underscores the overriding influence of physical nanotopography on stem cell functionality and indicates that the manipulation of biophysical cue presentation on substrate surfaces could be a method to precisely adjust cell behavior, which suggests that by fine-tuning the physical properties of substrates, researchers can guide stem cell differentiation and function, offering a promising approach for developing advanced biomaterials for cell response and cell therapies. A novel, contact-free micropatterning method was developed to meticulously craft the cellular morphologies of nanopillar arrays. This technique affords precise control over the shapes of cells and their nuclei across a variety of cell types, provides single-cell-level resolution, and supports the orchestrated spatial arrangement of cells and their organelles on nanotopographic surfaces [17]. This breakthrough harnesses the synergistic impacts of nanotopography-induced and cell shape-induced regulation of cellular behavior, thereby enabling the strategic programming of cell function and destiny through intricate and finely engineered microenvironments [18]. The research conducted by the Cooper team points to a dynamic process that could be significantly influenced by nanotopography at various phases of osseointegration [19]. This includes the initial modulation of immune responses, the recruitment of MSCs, and subsequent osteoblastic differentiation, culminating in the generation of the bone matrix and its mineralization. These findings suggest that nanoscale topography could positively direct the polarization of adherent macrophages toward anti-inflammatory and regenerative phenotypes while also fostering the osteoinductive phenotype of adherent MSCs [19]. These insights suggest that the topological features of a substrate may independently dictate the differentiation or destiny of stem cells, offering crucial insights into the mechanisms by which topological cues impact stem cell behavior.

The nuclear genome contains the majority of the genetic information essential for determining the characteristics of a cell, tissue, or organism, and it is intricately arranged in 3D space. This 3D genome architecture is crucial for genome functions such as transcription, which can characterize the nuclear microenvironment of individual genes in single-cell nuclei and can be related to their gene expression or other functional properties [20]. As a cellular mechanosensor, alterations in nuclear morphology are believed to have a direct impact on the structure of chromatin and the functionality of the genome, which in turn can dictate cell fate. While earlier research linked microtopography engineering to the efficient modulation of stem cell phenotypes, especially in terms of lineage-specific differentiation, by modifying chromatin conformation, direct evidence for contact-guidance-induced chromatin reprogramming and targeted cell differentiation by topography cues is limited. Research has demonstrated how micropillar patterns, via contact guidance, can induce changes in the nuclear and cellular morphologies of hBMSCs, thereby influencing the conformation of the cells’ chromatin and their osteogenic differentiation both *in vitro* and *in vivo*. In mice with critical-size cranial defects, implants featuring micropillar patterns that induce nuclear constriction were found to alter the chromatin conformation of the cells, enhancing bone regeneration without the requirement for external signaling molecules. These findings suggested that the topography of medical devices could be tailored to promote bone regeneration through chromatin reprogramming [21]. This aligns partially with our own findings [ 6, 12]. Although their study integrated ATAC sequencing and RNA sequencing, along with GO enrichment analysis, they did not identify specific genes that were differentially expressed as a result of topographic factors affecting chromatin accessibility.

YBX1 is recognized as a versatile protein that engages in an array of DNA- and RNA-dependent processes, including DNA repair and transcription, pre-mRNA splicing, mRNA stability and translation [22]. YBX1 has been reported to play an essential role in osteogenic progression. YBX1 has been reported to control the expression of pluripotency-related genes in embryonic stem cells [23]. YBX1 can also regulate multiple biological activities, including cell proliferation, differentiation, senescence, apoptosis, and tumor development [24]. Previous studies have reported a decrease in the expression of the splicing factor YBX1 in aging BMSCs in both mice and humans. The removal of Ybx1 from BMSCs in mice has been linked to accelerated bone loss, whereas its overexpression has been shown to stimulate bone formation. YBX1 is believed to orchestrate the osteogenic differentiation of BMSCs through the meticulous regulation of RNA splicing [15]. The lncRNA RAD51-AS1 enhances the proliferation and osteogenic differentiation of hBMSCs by interacting with *YBX1*, inhibiting the translation of Smad7 and Smurf2 and leading to the upregulation of PCNA and SIVA1 [25]. Moreover, *YBX1* influences the accessibility and reprogramming of chromatin. ATAC-seq of hepatic-specific *YBX1*-OE mice injected with CCl _4_ revealed increased chromatin accessibility compared with that in the CCl _4_-only group. The functional enrichment of OCRs in the *YBX1*-OE group indicated increased accessibility of pathways related to ECM accumulation, lipid purine metabolism, and oxytocin signaling [26]. In the context of translational regulation, YBX1 has been identified as a significant impediment to induced cardiomyocyte (iCM) induction. In a mouse model of myocardial infarction, the deletion of Ybx1 has been shown to enhance *in vivo* reprogramming, leading to improved cardiac function [27]. These findings suggest that YBX1 is involved in the regulation of chromatin accessibility and chromatin remodeling and can promote the osteogenic differentiation of BMSCs, which is consistent with our findings.

Furthermore, our findings demonstrated the effect of substrate topography on chromatin accessibility and the resulting gene expression in hBMSCs. We found that the topography of the electrostatic spinning surface promoted the osteogenic differentiation of hBMSCs by altering the chromatin accessibility of the promoter region of the *YBX1* gene and promoting the expression of YBX1. These discoveries partially shed light on the mechanotransduction pathway from the cytoskeleton to the nucleus, offering novel insights into stem cell regulation. Our results also suggest potential applications in regenerative medicine and tissue engineering, where the design of scaffolds with specific topographies could be used to direct stem cell differentiation towards desired lineages. This approach could provide a more controlled and predictable means of achieving tissue regeneration than traditional methods that rely on the inherent differentiation potential of stem cells alone.

Notably, the regulation of chromatin accessibility and gene expression by substrate topography is a complex process that likely involves multiple signaling pathways and regulatory mechanisms. Further studies are needed to elucidate the underlying mechanisms and identify additional factors that may fully influence the response of stem cells to topographic cues.

In conclusion, we have elucidated that the topographical features of these surfaces serve as a distinct modulatory elements, fostering the osteogenic differentiation of hBMSCs. This is achieved by influencing a range of cellular behaviors, including cell adhesion, spreading, morphology, pseudopod formation, and cytoskeletal organization, as well as by modulating the ECM-related processes. Through ATAC-seq and RNA-seq, our findings revealed that the nanotopography of electrospun surfaces promoted osteogenic differentiation by modulating the chromatin accessibility of the *YBX1* gene promoter, leading to its upregulation. Notably, the downregulation of YBX1 expression abrogated the influence of topographical cues on hBMSC osteogenesis, suggesting that YBX1 plays a crucial role in mediating the response to the surface topography of biomaterials. These insights contribute to a deeper understanding of the interplay between biomaterial properties and stem cell behavior, with implications for the design of biomaterials that promote desired stem cell fates in regenerative medicine applications. Our study underscores the importance of considering surface nanotopography in the design of biomaterials intended for the osteogenic differentiation of hBMSCs. The identification of YBX1 as a key mediator of this response opens up new avenues for the development of therapeutic strategies that harness the osteogenic potential of hBMSCs. By modulating the expression or activity of YBX1, it may be possible to enhance or direct the osteogenic differentiation of these cells at the molecular level. Furthermore, the methods and approaches employed in this study provide a framework for investigating the complex interactions between biomaterial properties and stem cell behavior, which could have broad implications for the field of regenerative medicine. Overall, our findings contribute to provide more nuanced evidence of the mechanisms underlying osteogenic differentiation and offer promising directions for future research and clinical applications.
